# Editorial: Organizing and financing universal primary health care systems – best practices and blueprints for low- and middle-income countries

**DOI:** 10.3389/fpubh.2024.1422873

**Published:** 2024-05-20

**Authors:** Ayan Jha, Wu Zeng, Marwa Farag, Allyala Nandakumar, Eduardo Gonzalez-Pier

**Affiliations:** ^1^Global Health Practice, The Palladium Group, Washington, DC, United States; ^2^Department of Global Health, School of Health, Georgetown University, Washington, DC, United States; ^3^School of Public Health, University of Saskatchewan, Saskatoon, SK, Canada; ^4^School of Economics, Administration, and Public Policy, Doha Institute for Graduate Studies, Doha, Qatar; ^5^Heller Graduate School, Brandeis University, Waltham, MA, United States

**Keywords:** primary health care (PHC), universal health coverage (UHC), sustainable development goals (SDGs), health system strengthening (HSS), health financing, human resources for health (HRH), low- and middle-income countries (LMIC)

In a world still recovering from the economic, health, and social disruptions of the COVID-19 pandemic, primary healthcare (PHC) is being reimagined as the cornerstone toward universal health coverage (UHC) through building more resilient health systems. Historically overlooked, PHC has gained renewed importance in post-COVID health system strengthening efforts as “*the most inclusive, equitable, cost-effective, and efficient approach*” to serve vulnerable populations (1). Comprehensive PHC can address 80–90% of lifetime population health needs (2), underscoring the imperative for countries to increase and optimize investments in their PHC systems (3, 4).

In this context, our Research Topic attempted to contribute to the understanding of how low- and middle-income countries (LMICs) can progress toward universal PHC systems that are organized and funded to meet population health needs while offering maximal financial risk protection. The article by Alegre et al. sets the tone for this Research Topic, arguing that despite more than 45 years of global efforts to enhance PHC, investments remain insufficient, and PHC systems are often weak and particularly unresponsive to the poor. The authors suggest five key strategies to transform PHC: integrating client-centered services, digitizing PHC, reinvesting efficiency gains, enhancing management practices, and boosting community engagement. Effective implementation of these strategies, tailored to specific contexts and aimed at sustainable health outcomes, is essential for reaching UHC and Sustainable Development Goals (SDGs) by 2030.

Three studies by Ogundeji et al., Alebachew et al., and Olago et al. present costing analyses to conclude that basic minimum packages of health services are still largely aspirational in Nigeria, Ethiopia, and Kenya respectively. In Nigeria, the per capita normative costs to deliver PHC services in Kaduna and Kano states were estimated to be 1.6–3 times the observed PHC service delivery costs. The ratio of per capita normative and average actual PHC costs ranged from 1.9 and 8.2 across nine regions in Ethiopia, and from 1.1 and 3.4 across six of the seven sampled sub-counties in Kenya. All three studies highlight the need to close the resource gap in PHC spending through a combination of increased domestic health expenditure, revised benefit packages, and efficiency gains through better designed provider payment mechanisms.

In Tanzania, Maiba et al. conducted a critical examination of two strategic purchasing mechanisms: a direct transfer of pooled donor funds from the federal government to PHC facilities (direct facility financing, DFF), and a results-based financing (RBF) scheme that incentivized service delivery. Both schemes exhibited efficiency in allocation and spending through well-defined benefit packages, contracting both public and private providers, and using output-based provider payment systems. The capitation-based DFF was more restrictive on how funds could be used, though it better utilized the public financial management system for monitoring and evaluation. RBF ensured a better purchaser-provider split, but required resource-intensive verification processes. Zhang et al. examined the impact on health expenditure of zero-markup drug policy (ZMDP), introduced from 2015 to 2017 in Shanghai, China. Sampling 150 public hospitals in Shanghai, the authors found that ZMDP, which aimed to reduce out-of-pocket health expenditure by lowering profits accrued in health facilities, did not decrease the total health expenditure. Despite reduced health expenditure on drugs due to the direct effect of ZMDP, hospitals could compensate for the loss by increasing earnings through prescribing more medical services. The study provides a critical lesson—well-intentioned reforms may not always bring desired benefits to the health system or potential beneficiaries due to health market failures, such as information asymmetry. On a different note, Getaneh et al. explored the satisfaction levels of beneficiary households of a community-based health insurance scheme providing primary, secondary, and tertiary care coverage in Legambo, Ethiopia. Nearly 60% of beneficiaries were satisfied, mainly as a result of shorter waiting periods, lower membership premiums, availability of laboratory and referral services, and expedited voluntary enrolment. These three articles provide guiding examples of how research can inform the improvement of ongoing health financing mechanisms targeting the PHC level.

Another group of articles focus on the innovative use of existing healthcare workforce or care delivery platforms to expand the population and service coverage of PHC. Mor, Ananth, et al. explore how LMICs can move away from traditionally physician-focused PHC delivery to one where community health workers (CHWs) play a more central role as comprehensive providers. Examining case studies from six global and Indian programs, the authors noted that close supervision, care coordination, defined referral pathways, medication management, proactive care, and cost-effectiveness were common factors for success. Further, they identify population empanelment, comprehensive assessment, risk stratification, use of defined care protocols, and cultural wisdom for community engagement as five essential elements for high-performance. In another article, Mor, Sen et al. explore if neighborhood pharmacies can be used as PHC delivery platforms in the South Asian context, examining four interventions from India and Bangladesh. While pharmacies excel in community orientation and first-contact care, shortfalls relate to providing continuity of care, family-centeredness, and cultural competency. Though promising, there is a need for additional training and tools to enhance pharmacies' capabilities for effective PHC delivery. Schiff et al. urge us to think about utilizing school-based platforms to jointly achieve better PHC and education outcomes in a post-COVID world, citing the renewed interest in WHO/UNESCO's Health Promoting Schools model. This requires structured collaboration between health and education ministries, and authors propose two pragmatic financing solutions for this partnership: an inter-ministerial joint financing mechanism, beginning with aligning budgets but evolving into a structured system for combining funds, and a fixed-term co-financing mechanism that leverages donor contributions to stimulate collaborative efforts.

With a fast-approaching deadline for SDGs and an emerging wealth of experiences on best practices across countries, we need to reflect on how to leverage cross-learnings to strengthen and repurpose PHC to accelerate progress toward UHC. We are excited to present this rich series of ten articles seen as blueprints for change on critical delivery strategies, cost dynamics, innovative workforce utilization, and policy implications, underscoring the urgency and complexity of transforming PHC systems to meet the health and financial protection needs of LMICs.

## Author contributions

AJ: Conceptualization, Writing—original draft, Writing—review & editing. WZ: Writing—original draft, Writing—review & editing. MF: Writing—original draft, Writing—review & editing. AN: Writing—original draft, Writing—review & editing. EG-P: Conceptualization, Writing—original draft, Writing—review & editing.
